# Hollow Fiber Coupler Sensor

**DOI:** 10.3390/s19040806

**Published:** 2019-02-16

**Authors:** Nithin Kuruba, Tao Lu

**Affiliations:** Department of Electrical and Computer Engineering, University of Victoria, Victoria, BC V8P 5C2, Canada; nithu.everyyear@gmail.com

**Keywords:** hollow core fiber, directional coupler, biosensor

## Abstract

We present a bi-conical optical directional coupler composed of solid and hollow core fibers. Through an evanescent wave coupling mechanism, the detection of liquid refractive index and its temperature are demonstrated. The experimental results illustrated that the sensor offers a sensitivity of 4.03±0.50 volts per refractive index units (V/RIU) for refractive indices ranging from 1.331±0.003 to 1.403±0.003 with a resolution of $3.5\times 10^{-3}$ refractive index units (RIU).

## 1. Introduction

Sensors play a crucial role in modern life. They improve the quality, reliability, and accuracy of the measurement systems that support the healthy living of a human being. They can capture physical parameters of a biological species or the concentration of biochemical species. To date, several optical sensors have been built upon optical fibers due to their excellent light-guiding capabilities. They offer high flexibility, easy multiplexity [1,2], and good sensitivity over existing systems. Some of the other advantages using optical fibers include low cost, small size [3], robustness, and the ability to be used in unfavorable conditions such as noise, electromagnetic fields [4], high voltages, nuclear radiation, and explosive or chemical corrosive media [5]. Their applications to humidity and moisture sensing are also reported [6].

Refractive index (RI) measurement is one of the key aspects for sensors, as identifying the right refractive index can label the samples and measure purity [7]. There are other applications that are associated with the measuring of RI and few among them are detecting salinity of water, adulteration of chemical liquids, determination of sugars, protein concentration, air pressure sensing, and environmental pollution [8,9,10,11,12]. The introduction of various refractometric techniques has spawned an evolution in RI sensing. RI can be measured by refractometers, which can be categorized to four main types, including traditional handheld refractometers, digital handheld refractometers, Abbe refractometers and inline process refractometers [7].

Many developments are carried out in building optical fiber-based RI sensors. These sensors measure the RI based on different optical fiber configurations and techniques. Some of them are based on Surface Plasmon Resonance (SPR) [13,14,15], Fiber Bragg Grating(FBG) [16,17,18,19], Long Period Grating (LPG) [20,21,22,23] and Fiber Coupling [24,25,26,27] techniques. Recently, hollow core fibers (HCFs) have been proved to be low cost, highly sensitive and feasible solutions for RI sensing and based on which some of the techniques have been outlined which employed HCFs such as hollow core Bragg fiber (HCBFs) [28,29], and Photonic crystal fiber (PCF) [30]. Hollow fiber has a hollow core and a solid cladding, typically made by fused silica. HCFs have been used not only for measuring refractive indices but also as a pressure sensor [31]. Their excellent geometrical structure makes them suitable for temperature sensing applications and few of them are developed [32,33,34].

In this article, we present a 2×2 optical fiber coupler with a solid core fiber (SCF) and HCF. The coupler was fabricated using hydrogen flame-based process [35,36]. HCF has a silica layer of RI similar to the cladding RI of a regular SCF, which imparts light-guiding capabilities. This property of the fiber enables it to be evanescently coupled with the light passing in SCF and in turn can be used as a sensing device to detect RI of various samples passed in core of HCF. In this device, the light is guided into and out of the device through the SCF. Consequently, light-coupling efficiency is high without any sophisticated alignment setup. On the other hand, the light only propagates in the coupling region of the hollow core fiber, which significantly reduces the transmission loss from the HCF. Therefore, this device maximizes its use of light power without introducing costly and sophisticated light alignment and sample injection structures.

In this paper, we used our fabricated coupler to sense the RI and consequently temperature of liquid sample flowing inside the core of HCF. This was possible because the amount of light evanescently coupled from the SCF to HCF and back to SCF depends on the RI of the flowing liquid. Meanwhile, the temperature change of the liquid changes its RI, and consequently the amount of light transmits to the SCF.

## 2. Results

### Experiment and Outcome

**Figure 1:**
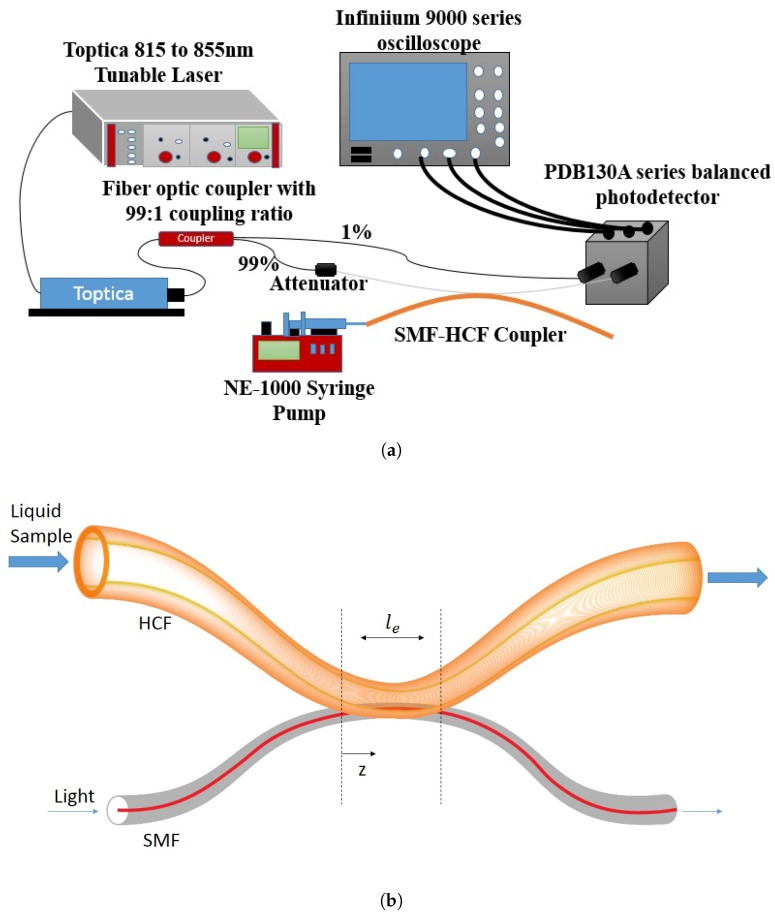
(**a**) Experiment setup for SCF–HCF fiber coupler; (**b**) Schematic drawing of the sensor.

The sensing experiment setup is shown in Figure 1a. A laser (Toptica DL Pro Tunable Laser) operating at around 850 nm wavelength is connected to one of the input ports of 2×2 fiber optic coupler with 99:1 coupling ratio. The 1% output branch is connected to a PDB100 series balanced photodetector. The 99% branch output port is connected to the SCF input port of our SCF–HCF coupler with a tunable attenuator in between. The SCF output port of our coupler further connects to the other input port of the balanced photodetector. The output voltage of the photodetector was recorded through Agilent DSO 9000 series oscilloscope driven by a custom built LabVIEW program. A regular 10 ml syringe driven by an NE 1000 programmable syringe pump was connected to the input port of the HCF to deliver the liquid sample under test to the coupler.

Using different attenuation settings, multiple tests were performed using blank Dulbecco’s Phosphate-Buffered Saline (DPBS). The transmission drop (ΔV) values corresponding to these tests obtained were 220.96 mV, 238.53 mV, 246.14 mV, and 217.02 mV; using these, the RI measurement resolution of the sensor was found.

In the next set of experiments, initially DPBS of RI = 1.331 was injected into HCF, the transmitted light at the output dropped suddenly as the solution crosses the coupling section and the change in the voltage is recorded. A least-square fit of the transmission spectrum to a step function indicates that the transmitted signal dropped to 432.17 mV from 750.89 mV, yielding a voltage drop ΔV of 320.40 mV. After recording the data, the DPBS was cleared from HCF by passing air through it. Later experiment trails included flowing of glycerol diluted in DPBS and recording the corresponding ΔV values.

**Figure 2:**
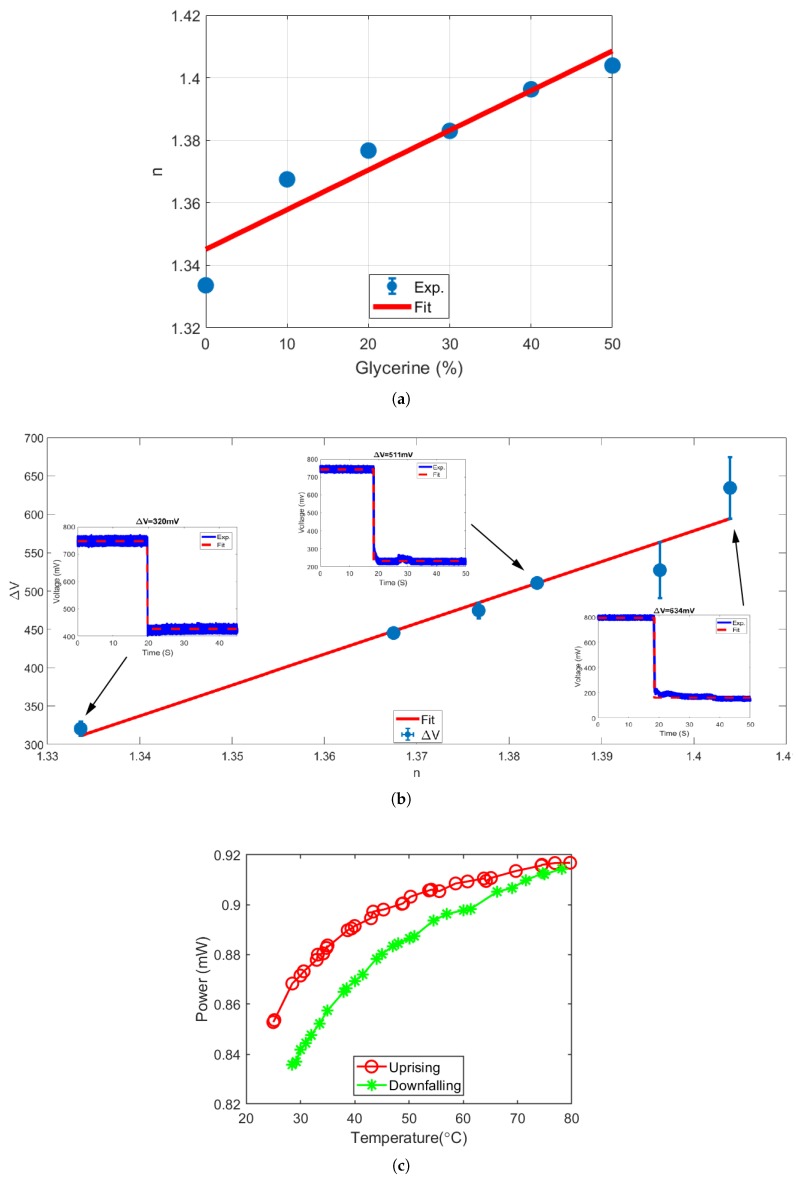
(**a**) Refractive index versus various concentration of glycerine solution in DPBS; (**b**) Output voltage versus the refractive index of glycerine/DPBS mixture; (**c**) Measured power in SCF as a function of temperature of the coupler filled with DI water.

The glycerol was used to prepare samples with different refractive indices. We prepared a total of six samples comprising of glycerol and DPBS in the percentage ratios of 0:100, 10:90, 20:80, 30:70, 40:60, and 50:50. We used a handheld pocket refractometer (Atago PAL-1) to calibrate the corresponding RI values for prepared samples and are plotted in Figure 2a.

HCF was cleaned with DPBS and infused with air after every experiment conducted using glycerol DPBS mixture to ensure HCF is free from residue molecules. Figure 2b shows a plot displaying all the ΔV values corresponding to change of glycerol percentage in the samples. The number of transmission drops recorded in a sequential set of experiments as a function of change in RI of samples show a clear linear relation in Figure 2b.

The relationship between the amount of transmission drop and RI of glycerol is investigated through regression analysis, in which the change in voltage (ΔV) is a dependent variable and RI (n) of glycerol is an independent variable. Due to the linear nature of the relationship, we considered a straight line and it is the simplest linear regression curve. The plot shown in Figure 2b also shows a residual or the error, which refers to the deviation between the linear curve and measured data. A minimum sensitivity of this sensor was estimated to be 4.02±0.50 V/RIU, through computing the slope of the linear curve. The precision value of 0.05 represents the error associated with the slope of regression line and to calculate it we initially calculate the standard error of estimate or root mean square error RMSE = 27.92 mV. Consequently, the error in slope was calculated to be 501.02 mV/RIU. The coefficient of determination $\left(R^{2}\right)$ of 0.9417 was calculated in our fitting, which shows that the measured values are very close to predicted values.

As the temperature of the liquid flow in the hollow core changes its RI, which in time changes the output power from the SCF, our coupler can operate as a temperature sensor. To demonstrate this, the fabricated coupler was placed on a hot plate. The hollow core region of HCF was filled with DI water. The temperature of hot plate was varied and Figure 2c shows all corresponding transmission power values recorded from SCF through a power meter.

## 3. Materials and Methods

**Figure 3:**
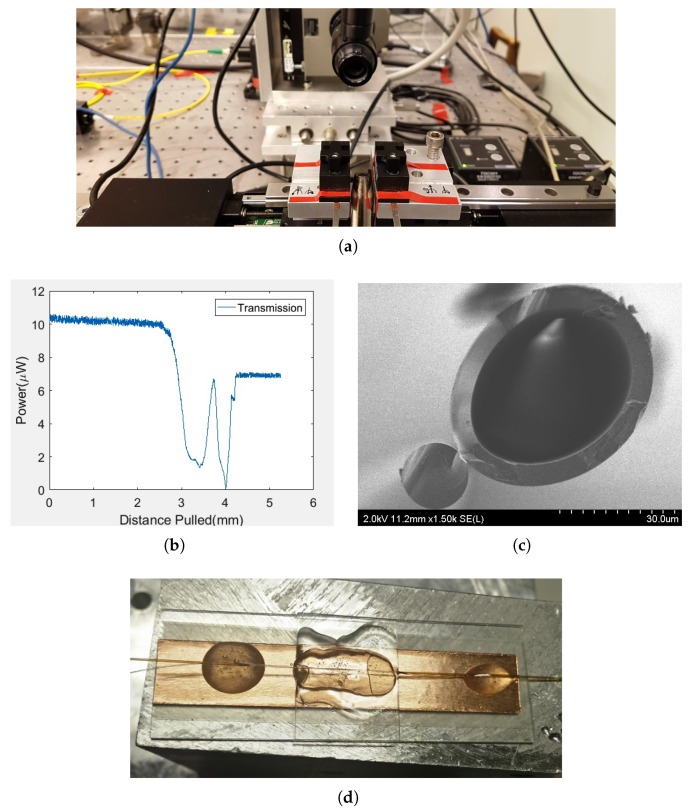
(**a**) Fiber-pulling setup holding fibers; (**b**) Transmission in SCF due to coupling of power in to HCF; (**c**) Cross section of SCF–HCF coupler; (**d**) Coupler staged on a microscopic glass slide and being held by quick epoxy.

In our experiment, we used SM980 as the SCF and polymicro capillary tubing (TSP320450) as the HCF. The diameter of the capillary tubing (HCF) inner core diameter is 320 ± 6 μm and its outer diameter is 435 ± 10 μm [37]. The coupler fabrication setup is as shown in Figure 3a. Both SCF and HCF fibers are twisted together near the coating free regions and the pair is mounted on the LabVIEW controlled motorized fiber-pulling stage. The hydrogen flame underneath points the twisted region of the pair, where the fibers are parallel to each other horizontally. During the fabrication, a laser source is connected to input of SCF and the output of SCF is connected to an optical power meter (Newport 1830c). Once the hydrogen flame is switched on, the twisted region of the pair gets heated up and softened, after which the motors are turned on. The motors, through the fiber clamps, pull the fiber pair in opposite directions. The coupling takes place between the two fibers as per the coupling principle stated in principle section. The output of the power meter is recorded through a data acquisition device (DAQ USB-6211). The recorded data is shown in Figure 3b, where the transmission power in SCF is plotted against the pulled distance. The SEM micrograph showing the cross section of the coupler is displayed in Figure 3c.

After building a SCF–HCF coupler, a microscopic glass with attached microheater was placed underneath the coupler. The PDMS solvent is prepared by carefully mixing part a and part b solvent in the ratio of 10:1. The PDMS solvent is dropped carefully onto the coupler and glass surface underneath. The microheater, placed below the glass slide, cures the PDMS solvent. The cured PDMS covering the entire coupler and the later ends up being glued with epoxy to the microscopic glass slide, as shown in Figure 3d.

## 4. Discussion

A detailed analysis [35,38] shows when the light is delivered to the input end of SCF, it will propagate along *z* direction with power transferring between SCF and HCF according to

(1) $$\begin{array}{ccc} \frac{P_{SCF}\left(z\right)}{P_{SCF}\left(0\right)} & = & 1-\frac{\kappa^{2}}{q^{2}}\sin^{2}\left(qz\right) \end{array}$$

(2) $$\begin{array}{ccc} \frac{P_{HCF}\left(z\right)}{P_{SCF}\left(0\right)} & = & \frac{\kappa^{2}}{q^{2}}\sin^{2}\left(qz\right) \end{array}$$

where $P_{SCF}\left(z\right)$ is the power after propagating at *z* distance from the input end of the SCF. $P_{HCF}\left(z\right)$ is that at HCF. *q* is defined as

(3) $$q=\sqrt{\kappa^{2}+\delta^{2}}$$

δ is the propagation constants mismatch between the modes of SCF and HCF with propagation constants $\beta_{SCF}$ and $\beta_{HCF}$ respectively and it is expressed as

(4) $$\delta =(\beta_{HCF}-\beta_{SCF})/2$$

The max power coupling efficiency is given as $\eta_{\max}$

(5) $$\eta_{\max}=(\frac{\kappa^{2}}{q^{2}})$$

Here κ is the mode coupling coefficient which describes how quickly the power exchange takes place between SCF and HCF. It can also be considered as the parameter that explains how efficiently the power couples from SCF to HCF and vice versa. According to [26], the coupling coefficient can be represented as,

(6) $$\kappa =\frac{\omega \epsilon_{0}}{4P_{0}}\int \int \left(n_{dc}^{2}\left(x,y\right)-n_{hcf}^{2}\left(x,y\right)\right){\hat{E}}_{scf}^{*}\cdot {\hat{E}}_{hcf}dxdy$$

in which, ω is the angular frequency of light, $P_{0}$ is unit power, $\epsilon_{0}$ is the permittivity of free space, $n_{dc}$ is the RI profile of SCF–HCF coupler, $n_{hcf}$ is the RI distribution of HCF, ${\hat{E}}_{scf}$ and ${\hat{E}}_{hcf}$ are the modal electrical field distributions with power normalized to unity.

**Figure 4:**
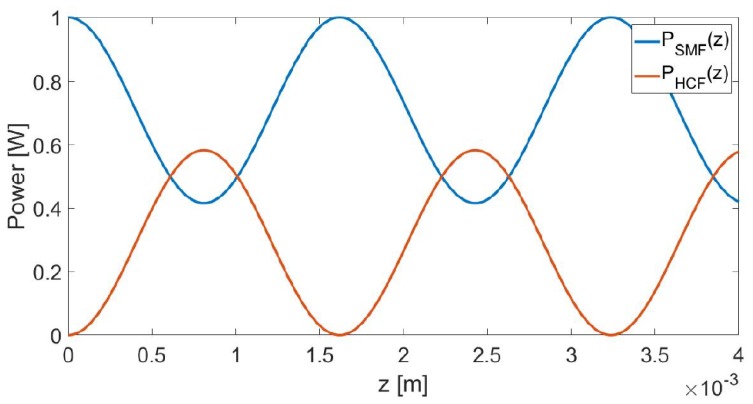
Exchange of optical power between SCF and HCF according to Equation (2).

The light power, after interacting with the test samples in the hollow core of HCF, will be transferred back to the SCF after reaching its maximum at $z=\frac{\pi }{2q}$ in HCF. Assuming the coupler has an effective coupling length $L_{c}$, the portion of light power exit the SCF output port will be $1-\frac{\kappa^{2}}{\gamma^{2}}\sin^{2}\left(qL_{c}\right)$. Noting that the output power is determined κ and δ, when liquid with slightly different RI flowing through the HCF core, the output power will be dominant by δ, which is approximately proportional to the liquid RI. Therefore, by monitoring the output power change, one may determine the RI of the liquid injected to the HCF. The transfer of light power between the two fibers along the propagation direction z is displayed in Figure 4 where the typical parameter values obtained from practical devices are adopted. Note the above analysis is based on the assumption that both SCF and HCF are in single mode. More accurate numerical modelling that incorporates multimode natures of the fibers can be performed using e.g., mode-matching methods [39,40].

## 5. Conclusions

In conclusion, we fabricated a SCF–HCF coupler sensor with simple PDMS packaging and demonstrated its applications in RI and temperature sensing. This sensing system is low cost, robust, portable, and sterilization-free. The sensor offers good sensitivity of 4.02±0.50 V/RIU, and a refractive index-sensing resolution of $3.5\times 10^{-3}$ RIU for refractive indices ranging from 1.331±0.003 to 1.403±0.003.

## Abbreviations

The following abbreviations are used in this manuscript:

SCF	Solid core fiber
HCF	Hollow core fiber
RI	Refractive index
